# Long-term effects on functional brain networks in adolescents treated for lumbar disc herniation

**DOI:** 10.1177/17448069251376189

**Published:** 2025-08-26

**Authors:** Sebastian Blomé, Granit Kastrati, Sebastian Pontén, Martin Jonsjö, Tobias Lagerbäck, Mikael Skorpil, Hans Möller, Maria Lalouni, Peter Fransson, Paul Gerdhem, William Hedley Thompson, Karin Jensen

**Affiliations:** 1 Department of Clinical Neuroscience, Karolinska Institutet, Solna, Sweden; 2 Department of Orthopedics and Hand Surgery, Uppsala University Hospital, Uppsala, Sweden; 3 Department of Clinical Science, Intervention and Technology (CLINTEC), Karolinska Institutet, Stockholm, Sweden; 4 Medical Unit Medical Psychology, Theme Women’s Health and Allied Health Professionals, Karolinska University Hospital Solna, Solna, Sweden; 5 Department of Physical Activity and Health, Swedish School of Sport and Health Sciences GIH, Stockholm, Sweden; 6 Department of Molecular Medicine and Surgery, Karolinska Institutet, Solna, Sweden; 7 Department of Neuroradiology, Karolinska University Hospital, Stockholm, Sweden; 8 Center for Spine Surgery in Stockholm, Stockholm, Sweden; 9 Department of Surgical Sciences, Uppsala University, Uppsala, Sweden; 10 Department of Applied Information Technology, University of Gothenburg, Gothenburg, Sweden

**Keywords:** Pain, neuroimaging, lumbar disc herniation, fMRI

## Abstract

Long-term effects of lumbar disc herniation treatment on brain function are poorly understood, and it is unclear when surgery should be recommended over non-operative treatment. The overall aim of the present study was to determine potential long-term effects on brain networks among individuals who received either surgical or non-operative treatment for lumbar disc herniation in adolescence. Brain network connectivity was assessed for individuals who received surgical treatment or non-operative treatment, and controls with no history of lumbar disc herniation. Prior to analysis, brain connectivity measures between groups were determined as main outcome, using functional magnetic resonance imaging. On average 12 years after treatment onset, the surgically treated cohort exhibited distinctly different functional brain connectivity, compared with both non-operative treatment and controls. The difference was neither attributed to self-reported pain, nor lumbar spine morphology. The findings suggest that surgical treatment for lumbar disc herniation in adolescence may be associated with a long-term imprint on the functional brain connectome.

## Introduction

As of 2020, long-term low back pain (LBP) remains the leading cause of years lived with disability globally.^1^ Neuroimaging studies show that long-term pain is associated with structural- and functional changes in the brain.^2,3^ Similarly, normalization of brain aberrations has been identified when long-term pain has been successfully treated, for example, in LBP,^4^ arthritis,^5^ and fibromyalgia,^6^ suggesting that brain abnormalities are not the cause, but a consequence of brain plasticity during persistent pain.

One cause for long-term LBP is lumbar disc herniation (LDH).^7^ LDH is common in adulthood, but rare in adolescence.^8^ LDH may be treated surgically or non-surgically.^9,10^ Non-surgical treatment consists mainly of medication, physiotherapy and lifestyle modifications, which has an equally large improvement rate in adults, although remission is often slower compared to surgery.^11,12^

Long-term outcomes of LDH treatments have previously been described in adults,^13–15^ but knowledge is still lacking when treatments are initiated during adolescence. Lagerbäck et al. previously performed a case/control study on the long-term effects of LDH surgery in adolescence, examining spinal Magnetic Resonance Imaging (MRI) characteristics as well as quality-of-life factors 14 years after treatment.^16^ Findings suggest that cases had more degenerative signs at the two lower lumbar levels (L4-L5 and L5-S1), more back pain and a lower quality of life compared to controls. In addition, Pontén et al. compared the same surgically treated cohort with individuals who received non-operative treatment and found similar degrees of lumbar degeneration and patient-reported outcomes at the time of diagnosis, and at long-term follow-up.^17^

Functional MRI (fMRI) of the brain, performed during a task free condition (so called resting-state fMRI), can be used to determine connectivity of intrinsic brain networks, referred to as the functional connectome, and allows for detection of network changes linked to disease. Long-term back pain,^18^ and spinal cord injuries,^19^ have previously been associated with altered connectivity in resting-state functional brain networks, but knowledge is lacking for long-term follow-ups. Over the last decade, researchers have utilized resting-state fMRI to investigate brain-related changes when suffering from LDH,^20^ and subsequent non-operative treatments.^21,22^ Using resting state fMRI and graph-based analyses, these studies suggest aberrant whole-brain network properties in LDH compared to controls.^20^ Also, preliminary evidence from an fMRI study suggests a possible change in the amplitude of spontaneous low-frequency fluctuations in the brain after treatment with manual therapy.^22^ To our knowledge, no brain fMRI studies have yet compared different treatment options for LDH.

Here, we used resting-state fMRI to determine the potential long-term effects on the functional connectome among individuals who received either surgical or non-operative treatment for LDH in adolescence, along with controls.

Three hypotheses were prepared to answer the research question.

**Hypothesis I:** There will be a significant difference in resting-state functional brain connectivity between the three cohorts: surgical, non-operative and control.
**Hypothesis II:** The brain region representing the lower back area in the primary somatosensory cortex will display cohort-specific resting-state functional connectivity profiles to the rest of the brain.
**Hypothesis III:** Severe intervertebral disc morphology, measured with the Pfirrmann grading score will correlate with functional connectivity measures in resting-state brain networks.

## Materials and methods

Ethical approval of this study was received from the Ethical Review Board in Stockholm (dnr: 2018/29931/1 and 2019-01713). The analyses were pre-registered and are available at OSF via https://osf.io/36qxc.The dataset had not been opened during the preregistration. Upon opening of the dataset, some changes in hypotheses were made before proceeding into analysis: Changes in hypotheses from preregistration included analyzing non-operative and surgically treated participants separated, and not pooled. Additionally, at the time of analysis there were no clinical cutoffs for a patient’s status of the lumbar back. Therefore, we used a pre-defined threshold of Pfirrmann score IV and above in the L4-L5 and L5-S1 lumbar back area to dichotomize participants to having “severe intervertebral disc degeneration” or not.^23^

### Participants

All participants gave oral and written informed consent. Exclusion criteria for all participants were: contraindications for undergoing MRI; serious health problems and/or any kind of substance abuse. An additional exclusion criterion for the non-operative cohort was a history of lumbar surgery.

Individuals treated surgically for LDH during adolescence were identified from the Swedish Spine register (SweSpine). Spinal MRI characteristics of participating individuals (*n* = 23) were previously compared with age- and gender matched controls (*n* = 23) to assess the long-term effects of surgery for LDH.^16^ Structural and resting-state functional brain MRI data at the time of follow-up (May 2019–Jan 2020) were available from all participants in both cohorts.

Individuals with non-operative treatment for LDH, diagnosed during adolescence, were identified based on hospital records at the Karolinska University Hospital Solna, Sweden. A total of 22 individuals were recruited and answered a follow-up survey. Two individuals were excluded because they had undergone surgery. Of the remaining 20 individuals, 14 underwent spinal MRI to compare their degree of degeneration to the degeneration at the time of diagnosis.^17^ Structural and functional brain MRI data were collected for 12 participants at the time of follow-up (Jun 2021 - Sep 2021) and were thus collected after the first two cohorts (May 2019 – Jan 2020) with no possibility to do a priori stratification on age and sex.

### Clinical characteristics

Clinically reported outcomes included disc degeneration assessment using the Pfirrmann classification on sagittal T2-weighted lumbar MRI images.^24^

Participants self-assessed the Oswestry Disability Index (ODI) survey, EQ VAS level, and Visual Analogue Scale (VAS) estimates of both back and leg pain.

ODI (score 0-100%) is a back-related disability score that ranges between 0% (no disability) to 100% (completely disabled).^25^

The EQ VAS (score 0-100) is a self-estimation of a person’s state of health. The score “100” represents the best possible condition and “0” the worst possible condition.^26^

The VAS (0-100 mm) was collected for last week’s back pain and leg pain. The anchor words “No pain” and “Maximum pain” was used for “0” and “100,” respectively.

### Image acquisition and preprocessing

Brain MRI data were collected with an 8-channel head coil and a 3.0T scanner (Discovery MR750, GE Healthcare) at MR-center, located in Karolinska University Hospital, Solna, Sweden. For acquisition of functional brain images, gradient echo-planar-imaging (EPI) was used with the following specifications: TR = 2205 ms, TE = 30 ms, flip angle = 80°, FoV = 230 × 230 mm, slices = 42, slice thickness = 3.0 mm^3^ (0.5 mm spacing, axial orientation, phase-encoding direction A/P), number of volumes = 403.

Collected brain images were evaluated in MRIQC,^27^ and then further preprocessed using fMRIPrep.^28^ A full description of the image preprocessing pipeline and steps from fMRIPrep are available in the supplementary materials. Motion correction and denoising of preprocessed functional brain imaging data were performed using the 36-nuisance regressor strategy.^29,30^ Subsequently, all images were band-pass filtered (0.008–0.1 Hz).

### Computation of functional brain connectivity

Preprocessed functional images were parcellated with FSL into regions of interest (nodes) using the Schaefer 100 atlas.^31,32^ The nodes were further grouped into seven brain networks defined in Yeo et al.^33^ Additionally, the Harvard-Oxford subcortical parcellation (13 nodes) and King 2019 cerebellum parcellation (10 nodes) were added.^34–38^ In total, nine brain networks were defined in the analysis. At a nodal level, all pairs were correlated with each other, producing a 123 × 123 nodal connectivity matrix for each individual. To address hypothesis II, we selected the two nodes covering the hip/trunk area of the somatosensory cortex (15 and 66 from the Schaefer/Yeo 100 parcel 7 network scheme) to conduct seed-based correlational analyses to the rest of the brain.

### Statistical analyses

Statistical analyses were conducted using MATLAB, Python and SPSS. Python scripts for analyses and illustrations are available at https://github.com/kipain/back2brain-connectivity.

Sex distribution between groups was compared using chi-square test. Age and clinical characteristics were compared between groups using ANOVA. Post hoc analyses were adjusted using two-sided Bonferroni correction with an alpha value of 0.05.

All brain connectivity matrices were fisher z-transformed prior to analysis. For cluster-based statistics addressing hypothesis I and III, a previously described Network-based statistic (NBS) approach was used to identify deviating clusters of connectivity patterns through permutation testing.^39^ This network-based approach corrects for multiple comparisons with family-wise error rate. The parameters used for the NBS analysis were f-statistics with a preregistered alpha of .05, an f-threshold of 9 (corresponding to a t-threshold of 3), and 10,000 permutations. *f_max_* represents the largest f-value found within a cluster. Post-hoc analyses used identical parameters.

The seed-based analyses addressing hypothesis II used FDR-corrected two-sided paired t-tests, comparing the mean differences in connectivity between seed nodes (left and right hemisphere, respectively) and all other regions of the brain.

## Results

### Clinical Outcomes

Demographics and clinical characteristics are summarized in Table 1. The differences observed in both age and sex for the non-operative cohort were taken into consideration by adding them as covariates in the brain network analyses. Chi-square test found no significant differences in the sex distribution (*x*^2^ = 4.521, df = 2, *p* = 0.1). There was however a significant difference in age between the three groups (*F*(2,63) = 22.4, *p* < 0.001).

**Table 1.** Demographics and clinical characteristics.

Variables	Surgery *n* = 23	Non-operative *n* = 20	Controls *n* = 23	*p*
Female (%)	12 (52%)	16 (80%)	12 (52%)	0.104
Age	31.3 (3.6)	24.9 (3.8)	31.3 (3.6)	<0.001
VAS Back pain	18.3 (20.6)	23.7 (23.0)	3.0 (5.2)	0.01
VAS Leg pain	8.0 (12.1)	12.6 (19.1)	3.1 (5.9)	0.07
ODI Score	11.7 (9.3)	13.3 (11.3)	1.5 (2.6)	<0.001
EQ VAS	80.9 (11.4)	73.7 (11.8)	90.0 (7.9)	<0.001

EQ: EuroQoL; ODI: Oswestry Disability Index; VAS: Visual Analogue Scale Displayed are the mean values (SD) and proportions (%). The VAS pain ratings (0–100) were collected as an average estimation from last week where 0 represents no pain and 100 the strongest imaginable pain. The ODI score ranges between 0% disability and 100% disability. EQ VAS is a self-estimation of the current state of health where 100 represents the best possible condition and 0 the worst possible condition.

Pain during the last week was compared between groups. A significant difference was found for pain in the back (*F*(2,62) = 7.6, *p* = 0.001), but not in the leg, (*F*(2, 62) = 2.7, *p* = 0.077). In post-hoc pairwise comparisons of back pain, we found that the control group reported significantly lower levels than both treatment cohorts, respectively (Surgery: *t*(43) = 3.4, *p* < 0.001, Non-operative: *t*(40) = 4.1, *p* < 0.001). However, there was no significant difference in back pain between the treatment cohorts (*t*(41) = -0.8, *p* = 0.425).

There was a significant difference between groups for the ODI score, (*F*(2,63) = 12.9, *p* < 0.001). Post-hoc pairwise comparisons presented a significantly lower disability score in the control group in comparison with both respective cohorts (Surgery: (*t*(44) = 5.1, *p* < 0.001, Non-operative: *t*(41) = 4.9, *p* < 0.001). For this comparison, no significant difference could be identified between the treatment cohorts regarding ODI score either (*t*(41) = −0.5, *p* = 0.621).

The EQ VAS for self-estimated health was significantly different between groups (*F*(2,62) = 11.1, *p* < 0.001). Pairwise comparisons revealed significantly higher health estimations in the control group when compared with surgical treatment (*t*(43) = -3.1, *p* = 0.003), as well as non-operative treatment (*t*(40) = −5.3, *p* < 0.001). Here, EQ VAS differed significantly between treatment cohorts with the surgical group reported higher health ratings (*t*(41) = 2.0, *p* = 0.048).

**Figure 1.**
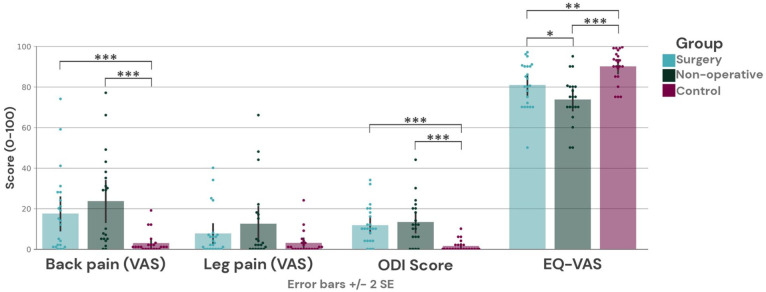
Self-reported outcomes. Error bars represent +/- 2 SE. (Sign. Code: <0.05 *, <0.01, ** <0.001 ***)

All significant comparisons survived Bonferroni correction at the alpha level of 0.05, two-sided, except for the significant difference in EQ VAS between surgical and non-operative treatment (Figure 1).

### Whole-brain connectivity

**Figure 2.**
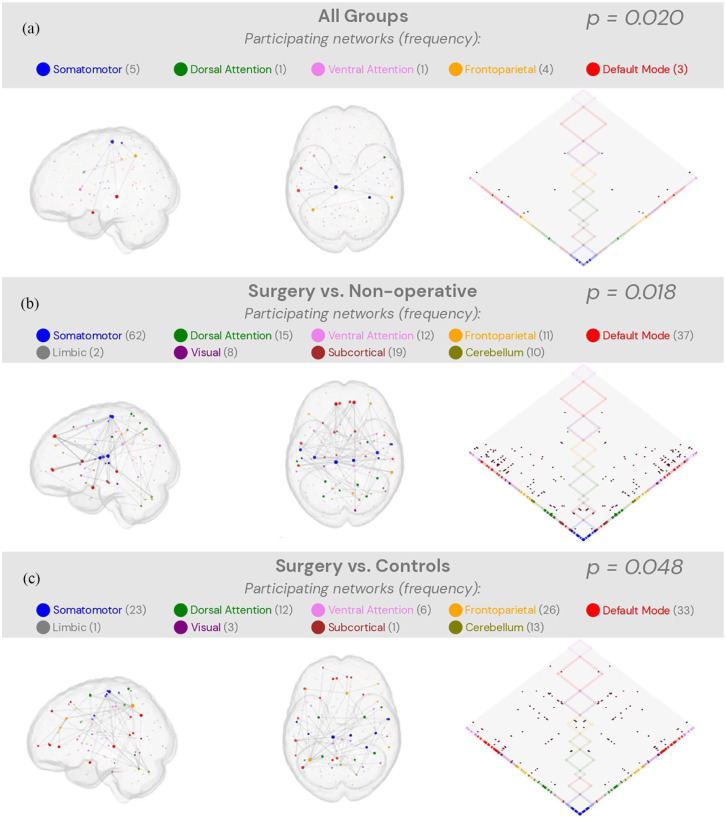
Significant brain network clusters. Permutation testing with F-statistics using the Network-based statistics generated three significant clusters where functional brain connectivity differed significantly (A-C). The size of the highlighted nodes from the cluster is synonymous with the number of connections it has. Frequency of participation for each network was obtained by counting the connections that reached their respective nodes.

One significant cluster of brain connectivity was detected, using permutation f-tests from NBS on all groups, after controlling for sex and age (Figure 2(a), *n* = 7 edges, *f_max_* = 11.44, *p* = 0.020). Post-hoc permutation f-tests for the three combinations of group pairs further found significant connectivity clusters when the surgical treatment cohort was compared with non-operative treatment (Figure 2(b), *n* = 88 edges, *f_max_* = 26.33, *p* = 0.018) and controls (Figure 2(c), *n* = 59 edges, *f_max_* = 20.74, *p* = 0.048). However, comparing non-operative treatment with controls resulted in no significant brain connectivity cluster (*p* = 0.35). The networks with the most prominent participations within these clusters were the somatomotor, frontoparietal, and default mode networks.

### Seed-based connectivity

**Figure 3.**
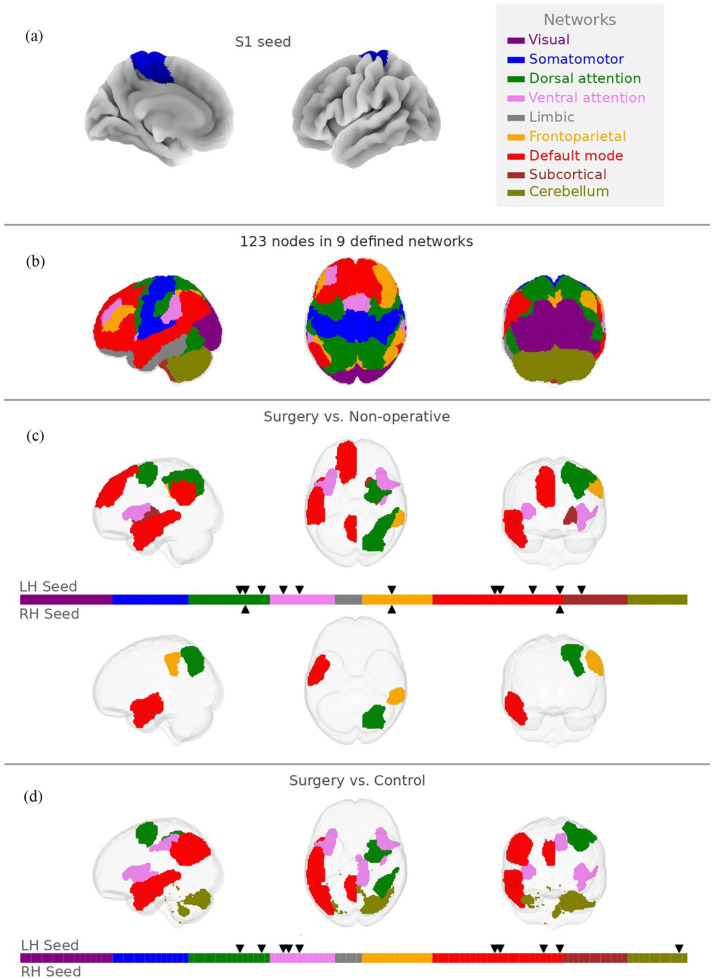
Seed-based connectivity analysis. (A) One node per hemisphere (LH/RH) that covered the back area of the primary somatosensory cortex was selected as seed region, illustrated in blue. (B) The connectivity with the remaining 122 nodes were pairwise compared. (C-D) Illustration of significantly different connections to seed between surgery/non-operative (C), as well as surgery/control (D) (p< 0.05, FDR). No significantly different seed connection could be identified by comparing non-operative treatment with controls, nor in the RH seed for the surgery/control comparison. Thus, no illustrations were generated.

The degree of connectivity between our *a priori* seed region of interest (S1 back area, Figure 3(a)) and the rest of the brain (Figure 3(b)) was different between the surgical group and the non-operative group at 14 significant connections (edges), divided on both right (RH) and left (LH) hemisphere (Figure 3(c)). All edges were significant after adjusting for 122 comparisons (nodes in rest of the brain) per analysis, Additionally, 10 edges to the LH seed were significantly different between the surgical group and controls (Figure 3(d)), but none were significant to the RH seed. No significant difference was observed between the non-operative group and controls after correcting for multiple comparisons.

### Brain connectivity and back morphology

A dichotomization based on severity of spinal morphology divided the participants into two equally large groups, pooled across all three groups. However, permutation testing saw no significant differences in brain connectivity.

## Discussion

In response to our first hypothesis, we found that a significant cluster of intrinsic brain connectivity separated the three groups (Figure 2). Pairwise statistical comparisons indicate that the main differences stem from the surgically treated cohort as no significant pairwise results could be identified for the non-operative versus control group. NBS clusters, derived from rejecting the null hypothesis that no differences exist between the groups compared, should be interpreted with caution as their properties may depend on other factors, such as signal-to-noise ratios and the chosen threshold parameter. Therefore, we will describe only the participating networks with a large proportion of connections across comparisons. These were the somatomotor, frontoparietal, and default mode network.

The somatomotor network represents primary somatosensory signaling and is therefore central for understanding pain processing, not necessarily in its own right but in connection to pain-related cognition and emotion circuitry.^40^ The frontoparietal network, including the prefrontal, parietal, and medial prefrontal cortices, is implicated in decision-making by integrating external information with internal representations.^41^ As such, the frontoparietal network plays a key role in pain modulation, with studies showing that anticipatory activity in this network predicts placebo responses.^42^ Notably, activation during pain anticipation overlaps with the resting-state frontoparietal network,^43^ suggesting its significant role in the internal representation of pain. In the present study, the differences in frontoparietal network connectivity between groups may reflect differences in long-term exposure to internal representation of pain, and related differences in neural imprint. In addition, a large number of studies demonstrate that pain affects the connectivity within the default mode network,^44–47^ and the degree of disruption has been linked to pain severity.^48^ In our data, the difference in brain connectivity between the surgical and non-operative group was likely not attributed to reported pain levels as the pain at follow-up was reported higher within the non-operative group (Figure 1).

For our second hypothesis, we determined the connectivity between the back area of the primary somatosensory cortex and the rest of the brain. These results furthered our previous findings of group differences reflected in connectivity between the somatomotor and default mode networks (Figure 3). Moreover, the dorsal- and ventral attention networks accounted for some significantly different connections to the somatosensory back seed as well. Both these functional networks are associated with salience signaling (importance attribution),^49^ but could not be linked to levels of perceived pain at follow-up, and are likely reflecting other aspects of group differences than current pain levels.

Lastly, we hypothesized that differences in spinal morphology would distinguish different functional networks in the brain. However, permutation testing of a group dichotomization, based on lumbar disc severity, did not yield any significant clusters of change within the brain. It has for long been suggested that MRI characteristics of the spine are not associated with pain levels and other behavioral outcomes,^50,51^ and our study further supports the proposal with a novel perspective by addressing functional brain connectivity.

A previous brain MRI study on chronic LBP used gray matter density to distinguish LBP patients from healthy controls with a 76% accuracy.^52^ Zhou et al. found similar networks to be activated for LDH patients in a resting-state brain fMRI study.^53^ Furthermore, they propose that changes in the visual network are associated with pain improvements in LDH when treated with Spinal Manipulative Therapy, a non-operative method.^53^ Du et al. have also examined the effects of Spinal Manipulative Therapy on LDH and suggested alterations in brain function, due to LDH, to be reversible using this treatment method.^21^ To our knowledge, no comparable studies exist for surgically treating LDH, which makes it difficult to address whether our findings support the reversibility when treated non-operatively or not. As our data did not contain baseline resting-state fMRI data, we were unable to address the involvement of the visual network in LDH improvement. However, the potential alterations in brain function does not appear to have been successfully reversed in the surgically treated group.

Overall, the non-operative cohort exhibited only a few significant differences in functional connectivity compared to controls. Contrastingly, the largest differences in brain connectivity were found between surgical and non-operative treatment. With the lack of baseline brain fMRI data, we are unable to further determine any if these differences and similarities hold any therapeutic value. If one would speculate, the surgically treated group may display these differences due to different baseline severities. It is common practice to recommend surgery for patients suffering from large disc herniations as they produce worse radiculopathies. If so, this would suggest that a long-term imprint in the functional connectome may have occurred due to a more severe baseline. Alternatively, it may occur when exposed to a higher degree of lumbar spine complications.

To a certain extent, our findings of group differences at 12-year follow-up contradict previous findings by Du, and Seminovicz et al., where altered functional brain connectivity following chronic low back pain was reverted upon successful treatment. However, unlike that population, the present study examines treatments during adolescence, in which a higher degree of cerebral plasticity may cause more pronounced functional reorganizations in response to back pain. This uncertainty could be resolved by replicating the procedure presented here on a population treated for LDH in adulthood and comparing the findings. Another aspect worth highlighting is that the non-operative treatment cohort has their spinal tissue intact. If the present differences between groups are the result from a long-term adaptation to an invasive procedure, it may be important to monitor such individuals further, to prevent adverse effects from developing. To shed light on the causality, future studies could characterize the functional brain connectome at baseline and compare the long-term outcome between a surgical and non-operative treatment.

Concerning the limitations of the study, we acknowledge the small sample size and urge for verifying replications in larger samples. Furthermore, without baseline functional brain images nor a comparable adult population, we are unable to address the causality of the changes presented here. Future longitudinally designed studies may elucidate whether the differences observed here are in fact imprints from plasticity in a surgically treated group. Lastly, we observed a heterogeneity within the controls as they exhibited varying degenerative signs of their lumbar discs and perceived pain.

To conclude, over a decade after treatment for LDH, we have identified a prominent change in brain communication patterns for those receiving surgery in their adolescence. Future investigations may elucidate whether these differences can be attributed to plasticity or perhaps an adverse effect from the intervention itself.

## Supplemental Material

sj-docx-1-mpx-10.1177_17448069251376189 – Supplemental material for Long-term effects on functional brain networks in adolescents treated for lumbar disc herniation Supplemental material, sj-docx-1-mpx-10.1177_17448069251376189 for Long-term effects on functional brain networks in adolescents treated for lumbar disc herniation by Sebastian Blomé, Granit Kastrati, Sebastian Pontén, Martin Jonsjö, Tobias Lagerbäck, Mikael Skorpil, Hans Möller, Maria Lalouni, Peter Fransson, Paul Gerdhem, William Hedley Thompson and Karin Jensen in Molecular Pain

## References

[1] Collaborators GBDLBP. Global, regional, and national burden of low back pain, 1990-2020, its attributable risk factors, and projections to 2050: a systematic analysis of the Global Burden of Disease Study 2021. Lancet Rheumatol 2023; 5: e316–e329. 10.1016/S2665-9913(23)00098-X PMC10234592 37273833

[2] Jensen KB Regenbogen C Ohse MC Frasnelli J Freiherr J Lundstrom JN . Brain activations during pain: a neuroimaging meta-analysis of patients with pain and healthy controls. Pain 2016; 157: 1279–1286. 26871535 10.1097/j.pain.0000000000000517

[3] Tracey I . Neuroimaging mechanisms in pain: from discovery to translation. Pain 2017; 158(Suppl 1): S115–S122. 10.1097/j.pain.0000000000000863 28141634

[4] Seminowicz DA Wideman TH Naso L Hatami-Khoroushahi Z Fallatah S Ware MA Jarzem P Bushnell MC Shir Y Ouellet JA Stone LS . Effective treatment of chronic low back pain in humans reverses abnormal brain anatomy and function. J Neurosci 2011; 31: 7540–7550. 21593339 10.1523/JNEUROSCI.5280-10.2011 PMC6622603

[5] Rodriguez-Raecke R Niemeier A Ihle K Ruether W May A . Structural brain changes in chronic pain reflect probably neither damage nor atrophy. PLoS One 2013; 8: e54475. 10.1371/journal.pone.0054475 PMC3566164 23405082

[6] Jensen KB Kosek E Wicksell R Kemani M Olsson G Merle JV Kadetoff D Ingvar M . Cognitive Behavioral Therapy increases pain-evoked activation of the prefrontal cortex in patients with fibromyalgia. Pain 2012; 153: 1495–1503. 22617632 10.1016/j.pain.2012.04.010

[7] Simon J McAuliffe M Shamim F Vuong N Tahaei A . Discogenic low back pain. Phys Med Rehabil Clin N Am 2014; 25: 305–317. 24787335 10.1016/j.pmr.2014.01.006

[8] Weber H . The natural history of disc herniation and the influence of intervention. Spine (Phila Pa 1976) 1994; 19: 2234–2238; discussion 2233. 7809761 10.1097/00007632-199410000-00022

[9] Lagerback T Elkan P Moller H Grauers A Diarbakerli E Gerdhem P . An observational study on the outcome after surgery for lumbar disc herniation in adolescents compared with adults based on the Swedish Spine Register. Spine J 2015; 15: 1241–1247. 25701544 10.1016/j.spinee.2015.02.024

[10] Schoenfeld AJ Weiner BK . Treatment of lumbar disc herniation: Evidence-based practice. Int J Gen Med 2010; 3: 209–214. 20689695 10.2147/ijgm.s12270 PMC2915533

[11] Truumees E . A history of lumbar disc herniation from Hippocrates to the 1990s. Clin Orthop Relat Res 2015; 473: 1885–1895. 24752913 10.1007/s11999-014-3633-7 PMC4418987

[12] Peul WC van Houwelingen HC van den Hout WB Brand R Eekhof JA Tans JT Thomeer RT Koes BW , Leiden-The Hague Spine Intervention Prognostic Study G. Surgery versus prolonged conservative treatment for sciatica. N Engl J Med 2007; 356: 2245–2256. 17538084 10.1056/NEJMoa064039

[13] Burkhardt BW Grimm M Schwerdtfeger K Oertel JM . The Microsurgical Treatment of Lumbar Disc Herniation: A Report of 158 Patients With a Mean Follow-up of More Than 32 Years. Spine (Phila Pa 1976) 2019; 44: 1426–1434. 31205183 10.1097/BRS.0000000000003113

[14] Cunha M Basto D Silva PS Vaz R Pereira P . Long-term outcome of redo discectomy for recurrent lumbar disc herniations. Eur Spine J 2023; 32: 534–541. 20230103. 36595137 10.1007/s00586-022-07493-4

[15] Chen BL Guo JB Zhang HW Zhang YJ Zhu Y Zhang J Hu HY Zheng YL Wang XQ . Surgical versus non-operative treatment for lumbar disc herniation: a systematic review and meta-analysis. Clin Rehabil 2018; 32: 146–160. 20170717. 28715939 10.1177/0269215517719952

[16] Lagerbäck T Kastrati G Moller H Jensen K Skorpil M Gerdhem P . MRI Characteristics at a Mean of Thirteen Years After Lumbar Disc Herniation Surgery in Adolescents: A Case-Control Study. JB JS Open Access 2021; 6 20211119. 10.2106/JBJS.OA.21.00081 PMC8613359 34841186

[17] Ponten S Lagerback T Blome S Jensen K Skorpil M Gerdhem P . Lumbar degeneration and quality of life in patients with lumbar disc herniation: a case-control long-term follow-up study. Acta Orthop 2024; 95: 92–98. 20240202. 38305634 10.2340/17453674.2024.39944 PMC10836153

[18] Baliki MN Geha PY Apkarian AV Chialvo DR . Beyond feeling: chronic pain hurts the brain, disrupting the default-mode network dynamics. J Neurosci 2008; 28: 1398–1403. 18256259 10.1523/JNEUROSCI.4123-07.2008 PMC6671589

[19] Hawasli AH Rutlin J Roland JL Murphy RKJ Song SK Leuthardt EC Shimony JS Ray WZ . Spinal Cord Injury Disrupts Resting-State Networks in the Human Brain. J Neurotrauma 2018; 35: 864–873. 29179629 10.1089/neu.2017.5212 PMC5863102

[20] Huang S Wakaizumi K Wu B Shen B Wu B Fan L Baliki MN Zhan G Apkarian AV Huang L . Whole-brain functional network disruption in chronic pain with disk herniation. Pain 2019; 160: 2829–2840. 31408051 10.1097/j.pain.0000000000001674 PMC6856436

[21] Du HG Wen Y Dong JX Chen S Jin X Liu C Ling DY Lv LJ . Brain plasticity following lumbar disc herniation treatment with spinal manipulation therapy based on resting-state functional magnetic resonance imaging. Heliyon 2024; 10: e37703. 10.1016/j.heliyon.2024.e37703 PMC11417269 39315226

[22] Wen Y Chen XM Jin X Ling DY Chen S Huang Q Kong N Chai JE Wang Q Xu MS Du HG . A spinal manipulative therapy altered brain activity in patients with lumbar disc herniation: A resting-state functional magnetic resonance imaging study. Front Neurosci 2022; 16: 974792. 36161170 10.3389/fnins.2022.974792 PMC9490403

[23] Teichtahl AJ Urquhart DM Wang Y Wluka AE O’Sullivan R Jones G Cicuttini FM . Lumbar disc degeneration is associated with modic change and high paraspinal fat content - a 3.0T magnetic resonance imaging study. BMC Musculoskelet Disord 2016; 17: 439. 27765024 10.1186/s12891-016-1297-z PMC5073831

[24] Pfirrmann CW Metzdorf A Zanetti M Hodler J Boos N . Magnetic resonance classification of lumbar intervertebral disc degeneration. Spine (Phila Pa 1976) 2001; 26: 1873–1878. 11568697 10.1097/00007632-200109010-00011

[25] Fairbank JC Pynsent PB . The Oswestry Disability Index. Spine (Phila Pa 1976) 2000; 25: 2940–2952; discussion 2952. 11074683 10.1097/00007632-200011150-00017

[26] Dolan P . Modeling valuations for EuroQol health states. Med Care 1997; 35: 1095–1108. 9366889 10.1097/00005650-199711000-00002

[27] Esteban O Birman D Schaer M Koyejo OO Poldrack RA Gorgolewski KJ . MRIQC: Advancing the automatic prediction of image quality in MRI from unseen sites. PLoS One 2017; 12: e0184661. 10.1371/journal.pone.0184661 PMC5612458 28945803

[28] Esteban O Markiewicz CJ Blair RW Moodie CA Isik AI Erramuzpe A Kent JD Goncalves M DuPre E Snyder M Oya H Ghosh SS Wright J Durnez J Poldrack RA Gorgolewski KJ . fMRIPrep: a robust preprocessing pipeline for functional MRI. Nat Methods 2019; 16: 111–116. 30532080 10.1038/s41592-018-0235-4 PMC6319393

[29] Satterthwaite TD Elliott MA Gerraty RT Ruparel K Loughead J Calkins ME Eickhoff SB Hakonarson H Gur RC Gur RE Wolf DH . An improved framework for confound regression and filtering for control of motion artifact in the preprocessing of resting-state functional connectivity data. Neuroimage 2013; 64: 240–256. 22926292 10.1016/j.neuroimage.2012.08.052 PMC3811142

[30] Ciric R Wolf DH Power JD Roalf DR Baum GL Ruparel K Shinohara RT Elliott MA Eickhoff SB Davatzikos C Gur RC Gur RE Bassett DS Satterthwaite TD . Benchmarking of participant-level confound regression strategies for the control of motion artifact in studies of functional connectivity. Neuroimage 2017; 154: 174–187. 28302591 10.1016/j.neuroimage.2017.03.020 PMC5483393

[31] Schaefer A Kong R Gordon EM Laumann TO Zuo XN Holmes AJ Eickhoff SB Yeo BTT . Local-Global Parcellation of the Human Cerebral Cortex from Intrinsic Functional Connectivity MRI. Cereb Cortex 2018; 28: 3095–3114. 28981612 10.1093/cercor/bhx179 PMC6095216

[32] Jenkinson M Beckmann CF Behrens TE Woolrich MW Smith SM . Fsl. Neuroimage 2012; 62: 782–790. 21979382 10.1016/j.neuroimage.2011.09.015

[33] Yeo BT Krienen FM Sepulcre J Sabuncu MR Lashkari D Hollinshead M Roffman JL Smoller JW Zollei L Polimeni JR Fischl B Liu H Buckner RL . The organization of the human cerebral cortex estimated by intrinsic functional connectivity. J Neurophysiol 2011; 106: 1125–1165. 21653723 10.1152/jn.00338.2011 PMC3174820

[34] King M Hernandez-Castillo CR Poldrack RA Ivry RB Diedrichsen J . Functional boundaries in the human cerebellum revealed by a multi-domain task battery. Nat Neurosci 2019; 22: 1371–1378. 31285616 10.1038/s41593-019-0436-x PMC8312478

[35] Makris N Goldstein JM Kennedy D Hodge SM Caviness VS Faraone SV Tsuang MT Seidman LJ . Decreased volume of left and total anterior insular lobule in schizophrenia. Schizophr Res 2006; 83: 155–171. 16448806 10.1016/j.schres.2005.11.020

[36] Frazier JA Chiu S Breeze JL Makris N Lange N Kennedy DN Herbert MR Bent EK Koneru VK Dieterich ME Hodge SM Rauch SL Grant PE Cohen BM Seidman LJ Caviness VS Biederman J . Structural brain magnetic resonance imaging of limbic and thalamic volumes in pediatric bipolar disorder. Am J Psychiatry 2005; 162: 1256–1265. 15994707 10.1176/appi.ajp.162.7.1256

[37] Desikan RS Segonne F Fischl B Quinn BT Dickerson BC Blacker D Buckner RL Dale AM Maguire RP Hyman BT Albert MS Killiany RJ . An automated labeling system for subdividing the human cerebral cortex on MRI scans into gyral based regions of interest. Neuroimage 2006; 31: 968–980. 16530430 10.1016/j.neuroimage.2006.01.021

[38] Goldstein JM Seidman LJ Makris N Ahern T O’Brien LM Caviness VS Jr. Kennedy DN Faraone SV Tsuang MT . Hypothalamic abnormalities in schizophrenia: sex effects and genetic vulnerability. Biol Psychiatry 2007; 61: 935–945. 17046727 10.1016/j.biopsych.2006.06.027

[39] Zalesky A Fornito A Bullmore ET . Network-based statistic: identifying differences in brain networks. Neuroimage 2010; 53: 1197–1207. 20600983 10.1016/j.neuroimage.2010.06.041

[40] Hashmi JA Baliki MN Huang L Baria AT Torbey S Hermann KM Schnitzer TJ Apkarian AV . Shape shifting pain: chronification of back pain shifts brain representation from nociceptive to emotional circuits. Brain 2013; 136: 2751–2768. 23983029 10.1093/brain/awt211 PMC3754458

[41] Vincent JL Kahn I Snyder AZ Raichle ME Buckner RL . Evidence for a frontoparietal control system revealed by intrinsic functional connectivity. J Neurophysiol 2008; 100: 3328–3342. 18799601 10.1152/jn.90355.2008 PMC2604839

[42] Wager TD Atlas LY Leotti LA Rilling JK . Predicting individual differences in placebo analgesia: contributions of brain activity during anticipation and pain experience. J Neurosci 2011; 31: 439–452. 21228154 10.1523/JNEUROSCI.3420-10.2011 PMC3735131

[43] Kong J Jensen K Loiotile R Cheetham A Wey HY Tan Y Rosen B Smoller JW Kaptchuk TJ Gollub RL . Functional connectivity of the frontoparietal network predicts cognitive modulation of pain. Pain 2013; 154: 459–467. 23352757 10.1016/j.pain.2012.12.004 PMC3725961

[44] Alshelh Z Marciszewski KK Akhter R Di Pietro F Mills EP Vickers ER Peck CC Murray GM Henderson LA . Disruption of default mode network dynamics in acute and chronic pain states. Neuroimage Clin 2018; 17: 222–231. 29159039 10.1016/j.nicl.2017.10.019 PMC5683191

[45] Ceko M Frangos E Gracely J Richards E Wang B Schweinhardt P Catherine Bushnell M . Default mode network changes in fibromyalgia patients are largely dependent on current clinical pain. Neuroimage 2020; 216: 116877. 32344063 10.1016/j.neuroimage.2020.116877 PMC8855626

[46] Jones SA Morales AM Holley AL Wilson AC Nagel BJ . Default mode network connectivity is related to pain frequency and intensity in adolescents. Neuroimage Clin 2020; 27: 102326. 32634754 10.1016/j.nicl.2020.102326 PMC7338779

[47] Loggia ML Kim J Gollub RL Vangel MG Kirsch I Kong J Wasan AD Napadow V . Default mode network connectivity encodes clinical pain: an arterial spin labeling study. Pain 2013; 154: 24–33. 23111164 10.1016/j.pain.2012.07.029 PMC3534957

[48] Napadow V LaCount L Park K As-Sanie S Clauw DJ Harris RE . Intrinsic brain connectivity in fibromyalgia is associated with chronic pain intensity. Arthritis Rheum 2010; 62: 2545–2555. 20506181 10.1002/art.27497 PMC2921024

[49] Fairclough SH Stamp K Dobbins C . Functional connectivity across dorsal and ventral attention networks in response to task difficulty and experimental pain. Neurosci Lett 2023; 793: 136967. 20221112. 10.1016/j.neulet.2022.136967 36379390

[50] Pfirrmann CW Metzdorf A Elfering A Hodler J Boos N . Effect of aging and degeneration on disc volume and shape: A quantitative study in asymptomatic volunteers. J Orthop Res 2006; 24: 1086–1094. 16609964 10.1002/jor.20113

[51] Witt I Vestergaard A Rosenklint A . A comparative analysis of x-ray findings of the lumbar spine in patients with and without lumbar pain. Spine (Phila Pa 1976) 1984; 9: 298–300. 6233717 10.1097/00007632-198404000-00014

[52] Ung H Brown JE Johnson KA Younger J Hush J Mackey S . Multivariate classification of structural MRI data detects chronic low back pain. Cereb Cortex 2014; 24: 1037–1044. 23246778 10.1093/cercor/bhs378 PMC3948494

[53] Zhou XC Wu S Wang KZ Chen LH Wei ZC Li T Hua ZH Xia Q Lyu ZZ Lyu LJ . Impact of spinal manipulative therapy on brain function and pain alleviation in lumbar disc herniation: a resting-state fMRI study. Chin J Integr Med 2025; 31: 108–117. 39707137 10.1007/s11655-024-4205-7

